# A review of computational drug repositioning: strategies, approaches, opportunities, challenges, and directions

**DOI:** 10.1186/s13321-020-00450-7

**Published:** 2020-07-22

**Authors:** Tamer N. Jarada, Jon G. Rokne, Reda Alhajj

**Affiliations:** 1 Department of Computer Science, University of Calgary, Calgary, Alberta Canada; 2 Department of Computer Engineering, Istanbul Medipol University, Istanbul, Turkey

**Keywords:** Computational drug repositioning, Drug repositioning strategies, Data mining, Machine learning, Network analysis

## Abstract

Drug repositioning is the process of identifying novel therapeutic potentials for existing drugs and discovering therapies for untreated diseases. Drug repositioning, therefore, plays an important role in optimizing the pre-clinical process of developing novel drugs by saving time and cost compared to the traditional de novo drug discovery processes. Since drug repositioning relies on data for existing drugs and diseases the enormous growth of publicly available large-scale biological, biomedical, and electronic health-related data along with the high-performance computing capabilities have accelerated the development of computational drug repositioning approaches. Multidisciplinary researchers and scientists have carried out numerous attempts, with different degrees of efficiency and success, to computationally study the potential of repositioning drugs to identify alternative drug indications. This study reviews recent advancements in the field of computational drug repositioning. First, we highlight different drug repositioning strategies and provide an overview of frequently used resources. Second, we summarize computational approaches that are extensively used in drug repositioning studies. Third, we present different computing and experimental models to validate computational methods. Fourth, we address prospective opportunities, including a few target areas. Finally, we discuss challenges and limitations encountered in computational drug repositioning and conclude with an outline of further research directions.

## Introduction

Drug repositioning has attracted considerable attention due to the potential for discovering new uses for existing drugs and for developing new drugs in pharmaceutical research and industry, due to its efficiency in saving time and cost over the traditional de novo drug development approaches [1, 2]. Drug repositioning is also known as drug repurposing, drug reprofiling, drug redirecting, drug retasking, and therapeutic switching.

At the present time, the drug repositioning approach has taken on a new urgency due to the worldwide Coronavirus disease (COVID-19) epidemic, which originated in China. The rapid onset of the epidemic and its potential for infecting large numbers of people (the reproduction number $R_0$ is greater than 1 in the absence of social distancing and other countermeasures) has led to an urgency for developing new drugs for dealing with this disease. The status of drug and vaccine development for COVID-19 is, therefore, rapidly changing and almost every day, there is an update of the state of the developmental effort [3]. Because of the urgency in developing new drugs and treatments traditional drug development is too slow and the faster repositioning approach has, therefore, attracted great interest due to its potential for finding drugs that could be used to combat the effects of the virus infection [4–6].

Generally speaking, traditional drug repositioning studies focus on uncovering drug effect and mode of action (MoA) similarities [7], revealing novel drug indications by screening the current pharmacopeia against new targets [8], investigating prevalent characteristics between drug compounds such as chemical structures and side effects [9], or discovering the relationships between drugs and diseases [10].

The explosive growth of large-scale biomedical and electronic health-related data such as microarray gene expression signatures, pharmaceutical databases, and online health communities that are publicly available along with high-performance computing has empowered the development of computational drug repositioning approaches that generally include data mining, machine learning, and network analysis [11]. Investigating the relationship between different biomedical entities forms a vital part of most recent studies in the drug repositioning field. These biomedical entities include drugs, diseases, genes, and adverse drug reactions (ADRs), etc.

**Fig. 1:**
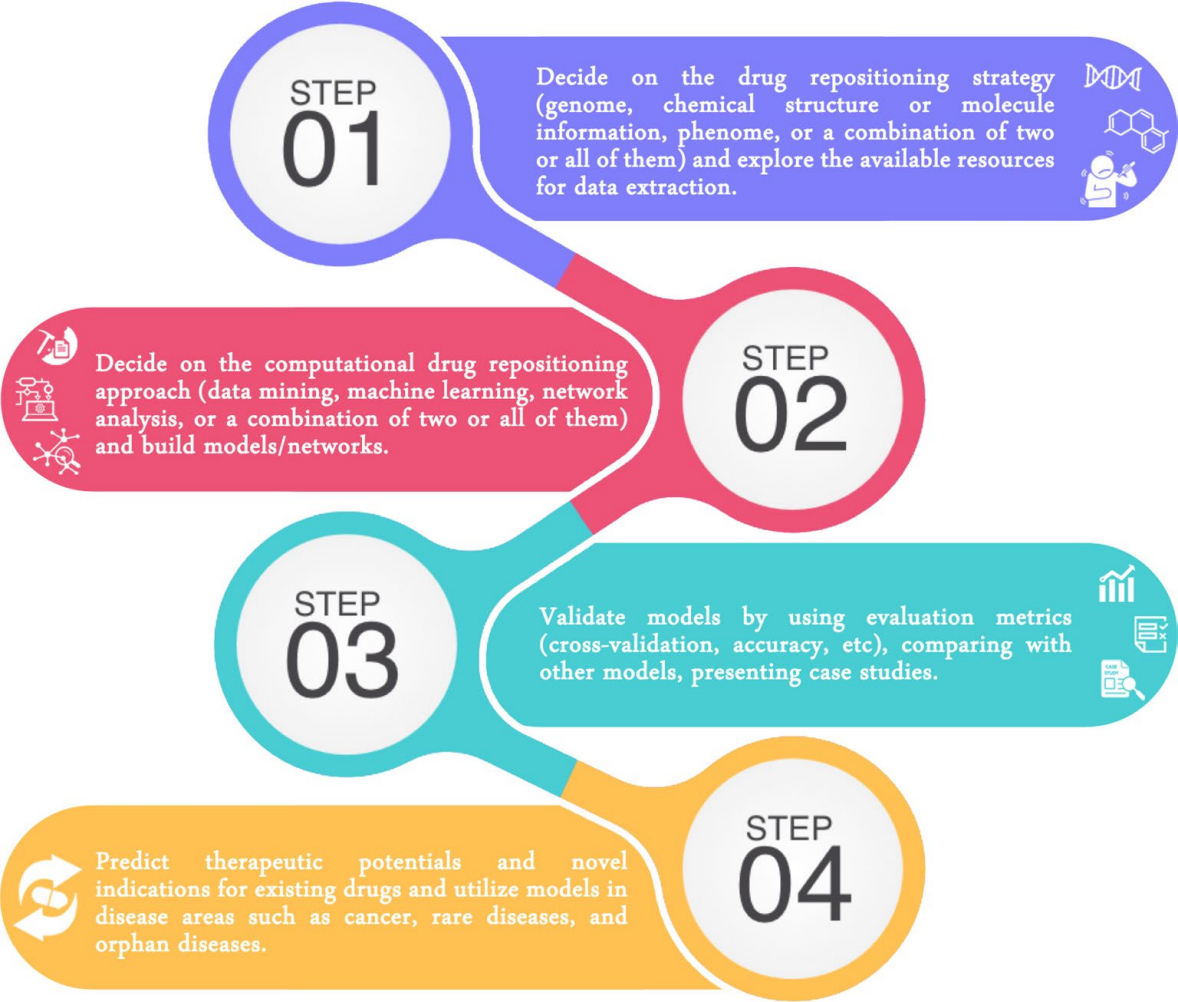
The workflow of computational drug repositioning studies

In this survey paper, we detail recent trends related to computational drug repositioning from various points of view. First, we recap different drug repositioning strategies and the corresponding data sources that are widely used. Second, we identify the computational approaches that are frequently used in drug repositioning studies. Third, we address computing and experimental validation models in computational drug repositioning research. Finally, we outline prospective opportunities, including a few target areas, and conclude with a summary of the outstanding complications and issues in drug repositioning. Figure 1 summarizes the workflow of computational drug repositioning studies, which, as shown, mainly comprise four main steps.

## Drug repositioning strategies

There are generally two fundamental drug repositioning principles. First, drugs related to a specific disease may also work on other diseases due to the interdependence between these different diseases. Second, a drug can be associated with various targets and pathways since drugs are confounding by nature [1]. Hence, drug repositioning studies could be classified into two categories based on where the findings originate from: (i) drug-based strategies where discovery originates from knowledge related to drugs and (ii) disease-based strategies where discovery originates from knowledge related to diseases.

### **Drug-based strategies**

Drug-based strategies depend on data related to drugs such as chemical, molecule, biomedical, pharmaceutical, and genomics information as the foundation for predicting therapeutic potentials and novel indications for existing drugs. Drug-based strategies are used where there is either substantial drug-related data accessible or significant motivation for studying how pharmacological characteristics can contribute to drug repositioning [10]. The vast majority of studies under this category share the hypothesis that if two drugs, $R_{1}$ and $R_{2}$ have similar profile and mode of action, and drug $R_{1}$ is used to cure disease *D*, then drug $R_{2}$ can be considered as a strong candidate for treating disease *D*. The two main strategies that represent this category are the genome strategy [7, 12–29], and the chemical structure and molecule information strategy [9, 30–39].

#### Genome strategy

A genome is a term that is used to describe all genes concerning a specific organism. In other words, biological data stored in a genome is represented by its DNA and is divided into separate units called genes [40]. The introduction of the human genome sequencing project [41] marked a turning point in the acquisition of knowledge at a molecular level about how living organisms function and revolutionized drug repositioning studies. More specifically, by finding the genes or proteins that perform a significant role in drugs and diseases’ molecular actions, the human genome sequencing project initiative has allowed a better understanding of drugs and diseases’ mode of actions. These genes and proteins have become enticing targets for governments and the pharmaceutical industry, which led to having this field of science as one of the most intensely studied research areas at research labs around the world.

The enormous volumes of publicly available genomic and transcriptomic data generated for disease samples, as well as clinical databases, provide a unique opportunity for understanding the disease and drug mechanisms of actions and discovering new uses for existing drugs. However, due to the tremendous complexity of biological systems, the comprehensive understanding of such systems is still incomplete. As a result, the research into a molecular explanation of biological systems is still pursued extensively.

It is noteworthy that the microarray gene expression profile is the most widely used transcriptomic profile among the genetic profile methods that have been explored for drug repositioning. Unlike most traditional molecular biology tools that allow the studying of a single gene or a small set of genes, microarray gene expression profiling captures the dynamic properties of a cell and measures all the transcriptional activity of thousands of genes at the same time, leading to a revolution in the molecular biology research field. The application of microarray gene expression profiling has, therefore, received considerable attention for its vital role in understanding how genes act at the same time and under the same conditions.

Computational drug repositioning studies using gene regulatory data presume that drugs target the same proteins with comparable gene expression profiles. This understanding has led to the discovery of a tremendous number of novel and unexpected functional gene interactions, the detection of novel disease subtypes, and the identification of underlying mechanisms of disease or drug responses [42–45].

The Connectivity Map (CMap) project and its extended Library of Integrated Network-Based Cellular Signatures (LINCS) are considered to be a key concept behind various well-recognized drug repurposing studies. The Connectivity Map can be defined as a combination of genome-wide transcriptional expression data that helps in revealing functional connections between drugs, genes, and diseases [12]. The extended project of the CMap produced large-scale gene expression profiles from human cancer cells that were targeted by various drug compounds in different environments [24, 28]. Lamb et al. [12] used microarray gene expression data to build a connectivity map that is used to discover relationships between the list of genes related to a specific disease or drug, called a query signature, and a set of gene expression profiles called the reference database. Expression profiles that are highly positively correlated to the query signature are considered to have a very similar mode-of-action to the query signature. Expression profiles that are highly negatively correlated to the query signature are considered for further treatment investigation.

Iorio et al. [7] developed an automatic approach that takes advantage of the similarity in gene expression profiles in order to discover drugs that have a shared effect and mode of action. Initially, the authors built a drug network where nodes represent drugs, and edges indicate similarities between a pair of drugs. Then, they used graph techniques for detecting drug communities. Drugs in each of these communities have a similar mode of action. Hu and Agarwal [13] conducted an extensive analysis of human drug perturbation and disease gene expression based on a negative correlation to construct a disease-drug network for predicting new applications for already approved drugs. Sirota et al. [14] performed a comprehensive systematic analysis of gene expression profiles for different diseases and drugs that led to discover new drug repositioning candidates.

CMap has gained considerable attention in drug repositioning since its introduction. It has shown promises in uncovering paths for drug repositioning for a variant group of diseases by identifying and suggesting new indications for existing drugs. Numerous researches have been conducted by integrating CMap data sources with other functional genomics databases such as the National Center for Biotechnology Information (NCBI) Gene Expression Omnibus (GEO) [46] to discover associations among genes, drugs, and diseases.

Jiang et al. [16] used CMap data sources to determine relationships between small molecules and miRNAs in human cancers in order to come up with therapeutic potentials and new indications for existing drugs. Jahchan et al. [21] used gene expression profiles to identify drug molecules for the treatment of small-cell lung cancer, which has not had effective treatments. Huang et al. [27] introduced a new connectivity map called (DMAP) that overcome the CMap data limitation by proposing a drug-protein connectivity map. DMAP consists of directed drug-to-protein effects and their scores. All previously-observed relationships between the associated drug and protein in various data sources were used to calculate effect scores from all database entries between the drug and protein as well as the confidence level of the quality of these calculated effect scores.

The massive amounts of publicly available gene expression profiles datasets have encouraged researchers to consider the guilt by association [47] concept to investigate drug–drug and drug–disease associations for identifying therapeutic indications for existing drugs. Iorio et al. [20] adopted the guilt by association concept to compare different drugs in order to identify any transcriptional responses similarity assuming that these drugs would share a similar mode-of-action (MoA).

Recently, microRNAs (miRNAs) have received considerable attention in biological and biomedical studies for their roles in regulating different types of cell activities [25, 26]. Hence, miRNAs have become key players in identifying drug repositioning therapeutic targets since miRNAs are vital for homeostasis of cells and active in many disease stages [48].

Jiang et al. [16] also used miRNAs along with small molecules, as potential drugs, to build networks for different types of cancer in order to identify small molecule-miRNA associations for drug repositioning based on miRNA regulations and transcriptional responses. There have been several attempts to build public repositories aiming to elevate the development of small molecule-based miRNA therapeutics.

Liu et al. [15] manually curated scientific literature looking for how small molecules affected miRNA expressions and developed a database (SM2miR) in order to capture existing small molecule-miRNA associations aiming. Li et al. [22] manually retrieved experimentally supported miRNA-disease associations from scientific articles and built the Human MicroRNA Disease Database (HMDD v2.0) to facilitate data exploration. Huang et al. [29] introduced (HMDD v3.0) by adding a significant number of miRNA-disease associations to (HMDD v2.0) and improving the accuracy of these associations based on literature-based evidence. Rukov et al. [19] established a database (Pharmaco-miR) to identify miRNA-gene-drug triplet set associations by combining data on miRNA targeting and protein-drug interactions.

Meanwhile, as most of the genome-based studies have focused on using gene expression profiles as a valuable source for discovering therapeutic indications for existing drugs, some studies have focused on other different types of genomic profiles such as genome-wide association studies (GWAS). GWAS follows the phenotype-to-genotype concept, where it starts with a specific genotype and checks for associations with genetic variants across the genome [49].

Sanseau et al. [17] filtered published GWASs catalog of disease-associated genes to come up with a list of GWAS-associated genes that were then evaluated against targets of drugs under clinical and preclinical investigation for potential novel indications and drug repositioning opportunities. Okada et al. [23] developed a new in silico approach by conducting a multi-stage GWAS analysis of targeted disease patients to uncover a set of unknown risk loci related to the targeted disease and further identify a set of biological candidate genes that are targeted by already approved drugs. Also, a collection of existing drugs approved for other indications was identified and linked to the studied disease for potential drug repositioning chances.

Garnett et al. [18] conducted a large-scale multivariate analysis of genetic of cancer cell lines and drugs in pharmaceutical pipeline projects to unveil new biomarkers of sensitivity and resistance to cancer therapeutics. To a certain degree, mutated genes demonstrate the molecular activity of drugs and can be considered as drug biomarkers during the drug repositioning process. A few mutated cancer cell line genes were found to be associated with drug sensitivity, which may serve as potential biomarkers for drug repositioning.

#### Chemical structure and molecule information strategy

As the genome drug-based strategy assumes drugs share common indications because of having similar profiles, chemical structure and molecule information of drugs are also considered to be worthwhile sources of pointing towards any transcriptional responses similarity between drugs for repositioning opportunities as these drugs usually affect genes, proteins, and other biological entities in similar forms [33, 34, 36]. Chemical structure similarity can be measured in various ways, such as two-dimensional (2-D) topological fingerprints and three-dimensional (3-D) conformational fingerprints [50].

Keiser et al. [9] proposed a systematic chemical structure similarity approach to screen compounds of existing and in-process drugs against hundreds of ligands that bind protein targets. Chemical structure similarity between drug compounds and ligand targets revealed thousands of unforeseen associations, some of which were tested and confirmed experimentally. The proposed approach can explain some of the side effects of existing drugs, and may also contribute to the identification of new repositioning applications for existing drugs.

Swamidass [33] suggested using chemical structures to determine which drug targets would modulate disease-relevant phenotype. Such a tactic would give indications of how other drugs, with similar chemical structures, modulate disease-relevant phenotype and hence treat the disease.

Most frequently, chemical structure similarity is incorporated with molecular activity and other biological information to identify new associations and potential off-target effects for approved and investigational drugs. Yamanishi et al. [30] developed a supervised learning model for a bipartite graph to identify possible drug-target interactions. The authors integrated drug chemical structure information, protein-protein interaction network, and drug-target interaction network to predict therapeutic potentials and unveil drug repositioning applications. Kinnings et al. [31] used drug chemical interactions under different environment variables to build a drug similarity network where drugs are defined as nodes, and an edge is drawn between two drugs when they have a high similarity score. Then, the authors analyzed the drug network to detect different drug communities and investigated drugs within each community for potential drug repositioning. Bleakley et al. [32] proposed a statistical method to predict drug-target interactions using chemical structure information and genomic sequence information. The authors built a supervised learning bipartite graph model based on independent local supervised learning problems to predict target proteins of a given drug and then to predict drugs targeting a given protein.

Li and Lu [35] combined drug chemical structure information with drug targets and interactions information to develop a novel bipartite graph model to calculate drug pairwise similarity. The results significantly enriched both the biomedical literature and clinical trials when compared to a control group of drug uses. The developed approach outperformed other approaches that only use drug target profiles and captured the implicit information between drug targets. Wang et al. [37] integrated drug chemical structure date along with molecular activity and drug side effect data to check for drug similarity and predict drug-diseases interactions.

Tan et al. [38] came up with a new form of “expression profile” based on 3-D drug chemical structure information, gene semantic similarity information, and drug-target interaction networks. The authors gave consensus response scores (CRS) between each drug and protein and used the absolute value of correlation coefficient between every two drugs as their degree similarity to build a drug similarity network (DNS), which led to identifying new drug indications. The proposed approach took into consideration the 3-D drug chemical structure information to overcome the instability of gene expression profiles acquired from different experiments due to experimental conditions such as environment and patient age.

While most of the available drug repositioning approaches that use chemical structure strategy focus on predicting direct or indirect drug interactions on a small scale, Zheng et al. [39] conducted a large scale study on drug-target relationships and introduced a new algorithm called Weighted Ensemble Similarity (WES). The authors identified the key ligand structural features of a protein as a set named ensemble. Rather than comparing two compounds to determine their similarity, each compound was compared to ensembles in order to calculate the overall ensemble similarity instead of using a single ligand similarity because ensembles usually represent smaller chemical structure features. The whole ensemble similarity scores were normalized and used to predict direct interactions of drugs and targets.

A further molecular repurposing strategy is provided by the geometry of a drug molecule as expressed by the proteomic signature. That is, repurposing candidates are identified by their proteomic signature similarities. This approach is exploited by Mangione and Samudrala in the paper [51] which describes a simulation system for drug molecule docking interactions applied to the repurposing of drugs. The shapes of molecules are in general determined using X-ray diffraction techniques and recent advances in the type of molecular docking required for the repurposing are discussed by Yan et al. [52] where the general limitations of the approacher for molecular shape determinations are also outlined.

### **Disease-based strategies**

Disease-based strategies depend on data related to diseases such as phenotypic traits information, side effects, and indications information as the foundation to predict therapeutic potentials and novel indications for existing drugs. Disease-based strategies are used when there is either insufficient drug-related data available or when the motivation in studying how pharmacological characteristics can contribute to drug repositioning effort concentrated on a particular disease [1]. The studies under this category share the hypothesis that if two diseases, $D_{1}$ and $D_{2}$ have a similar profile and indications, and drug *R* is used to cure disease $D_{1}$, then drug *R* can be considered as a strong candidate for curing disease $D_{2}$. The primary strategy that represents this category is the phenome strategy [10, 53–60].

#### Phenome strategy

The phenome is described as the overall set of phenotypic traits information, and it has arisen as a new strategy to connect drugs with clinical effects for drug repositioning due to the argument that it represents the unwitting effects of a drug and defines the physiological consequences of its biological activities. Moreover, the phenotypic expression of a drug’s side effect may be closely related to the phenotypic expression of a disease, which suggests that both the drug and the disease may share similar underlying pathways [10].

Clinical side effects and unexpected activities derived from off-targets have been shown to have the ability of profiling human phenotypic traits related to drugs and may ultimately help unveil potential therapeutic uses for these drugs. Campillos et al. [53] proposed a side-effect similarity measure based on the strong correlation between targeted portion binding profiles and side-effect similarity and experimentally verified that side-effect similarity indicates novel therapeutic uses for existing drugs. Yang and Agarwal [54] demonstrated that clinical side effects could be used to build a phenotypic profile of drugs and identify potential new disease indications. A side effects-drugs relationship dataset was integrated with a drug-disease relationship dataset to derive side effects-disease relationships. Then, side effects were used as features for building a prediction model for disease indications.

Ye et al. [57] constructed a drug–drug similarity network based on clinical side effects assuming that drugs with similar side effects may share similar therapeutic indications. Novel drug indications were identified in addition to already known indications. Bisgin et al. [58] used side effect information to build a model for predicting new therapeutic indications for existing drugs. It is worth mentioning that a profound background in molecular mechanisms is required for using phenotypic traits information in predicting new drug indications. While most of the phenotypic based research is leveraging data from clinical studies and drug labels, Nugent et al. [59] used side-effect data mined from social media to identify novel therapeutic indications in addition to previously identified indications.

Eventually, phenotypic traits information can be integrated with other data sources such as genome for therapeutic potentials and novel drug indications. Hoehndorf et al. [55] used phenotypic similarity to identify genotype-disease associations which were later combined with genotype-disease association data to predict novel drug-disease associations. Such a model can be considered as an introduction to an integrated system to identify drug-disease associations for diseases with an unknown molecular basis. Gottlieb et al. [56] developed a model using various drug–drug similarity measures, including phenome-based similarity, to predict novel drug–drug interactions and severity level associated with each of these interactions. Sridhar et al. [60] integrated different drug–drug similarity measures, including phenotypic similarity with already known drug–drug interactions to unveil drug–drug interactions, including several novel interactions.

### **Data resources**

The advanced technologies nowadays have produced a massive amount of data (e.g., gene expression, drug-disease associations, drug chemical structure profiles, drug targeted proteins, phenotypic traits), which has supported the enormous effort that has been devoted towards developing fascinating drug repositioning strategies. A list of the widely used data resources and their drug repositioning strategies classification is summarized in Table 1.

**Table 1:** Data resources widely used in drug repositioning research

Drug repositioning strategy	Data resource	Description (as of January 2020)
Genome	ArrayExpress Ê^61^]	A repository of high-throughput functional genomics experiments data
	Cancer Cell Line Encyclopedia (CCLE) [62]	A combination of gene expression, chromosomal copy number, and massively parallel sequencing data from human cancer cell lines
	Database for Annotation, Visualization and Integrated Discovery (DAVID) [63]	A comprehensive set of functional annotation tools
	Drug versus Disease (DvD) [64]	A platform for comparing drug and disease gene expression profiles retrieved from publicly available microarray data resources
	Expression Atlas [65]	A tool to explore gene expression data across species and different biological conditions
	Gene Expression Omnibus (GEO) [66]	A repository of gene expression profiles
	Gene Set Enrichment Analysis (GSEA) [67]	A tool for interpreting gene expression data
	Gene Signature Database (GeneSigDB) [68]	A repository of gene signatures data reported in the literature
	Gene Ontology (GO) [69]	A repository of functional genomics
	International Cancer Genome Consortium [70]	A repository of genomic data for many cancer types
	Kyoto Encyclopedia of Genes and Genomes (KEGG) [71]	A repository of genome sequencing and high-throughput functional genomics experiments molecular datasets
	Library of Integrated Network-based Cellular Signatures (LINCS) [72]	A catalog of gene expression data and how human cells respond to different genetic and environmental conditions
	Molecular Signature Database (MsigDB) [73]	A repository of annotated gene sets and expression profiles
	The Cancer Genome Atlas (TCGA) [74]	A repository of genomic data for more than 30 cancer types
	The Connectivity Map (CMap) [75]	A collection of genome-wide transcriptional expression data
	The Universal Protein Resource (UniProt) [76]	A repository of protein sequence and functional information
Phenome	ClinicalTrials.gov [77]	A repository of publicly and privately funded clinical studies from around the world
	Side Effect Resource (SIDER) [78]	A repository of adverse drug reactions related to drugs. Information includes drug side effects and side effects classifications
Chemical structure	ChEMBL [79]	A repository of drug structural information, such as 3D structures, and abstracted biological activities, extracted from the scientific literature
	Chemicalize [80]	A repository of chemical structure information
	ChemSpider [81]	A repository of chemical structure information
	DrugBank [82]	A repository of drug-related information, such as chemical structure for drugs
	Drug Central [83]	A repository of drug-related information such as chemical structure and biological activities
	PubChem [84]	A repository of chemical substances and their biological activities
	Protein Data Bank (PDB) [85]	A repository of 3D shape of proteins and nucleic acids information
	SWEETLEAD [36]	A repository of 2D and 3D drug chemical structure information, including approved and illegal drugs
	The NCGC Pharmaceutical Collection (NPC) [86]	A collection of chemical structure information related to approved and investigational drugs
	Therapeutic Target Database (TTD) [87]	A repository of drug-related information such as 3D structure, therapeutic class, and clinical development status
Phenome/genome	repoDB [88]	A repository of approved and failed drug-disease associations
	Online Mendelian Inheritance in Man (OMIM) [89]	A repository of human genes and genetic phenotype information
	The Pharmacogenetics and Pharmacogenomics Knowledge Base (PharmGKB) [90]	A repository of drug-related information such as drug labels, drug-gene associations, and genotype-phenotype relationships
Phenome/chemical structure	Drugs@FDA Database [91]	A repository of FDA approved drugs and related information

## Computational drug repositioning approaches

A significant challenge in drug repositioning is to distinguish between the molecular targets of a drug and the hundreds to thousands of additional gene products that respond indirectly to changes in the activity of the targets. Unfortunately, classical statistical approaches are ineffective for detecting the molecular targets of a drug among the vast amount of genes. Moreover, conventional statistical methods use small datasets and biological networks that are coming from experiments on different platforms and environments, which might lead to inconsistent findings reported by some studies. Also, when the data used to conduct such studies is limited, or the biological network is small, the proposed approaches might recover only partial knowledge of a living system. As a result, some approaches that claim inferences and discoveries may not be replicated.

The amount of publicly available large-scale biomedical and pharmaceutical data is growing exponentially, and computational drug repositioning approaches using data mining, machine learning, and network analysis become ever more critical when it comes to systematic drug repositioning due to the ability to overcome classical statistical approaches limitations and unreliable conclusions.

The drug repositioning field can benefit from new computational methods in detecting relationships among different types of biological entities such as genes, portions, diseases, and drugs and identify therapeutic potentials and novel indications for existing drugs. Such findings would help to treat cancer and other incurable illnesses, which eventually require the necessary and sufficient data to undertake the intended research. Table 2 presents an overview of computational drug repositioning studies, the adopted strategies, computational approaches, main techniques, data sources, key findings, and evaluation metrics.

### Data mining

The tremendous amount of genes, drugs, and diseases related information stored in databases in addition to the vibrant literature grown by the rapid increase in the number of the biological, biomedical, and pharmaceutical studies have led to the need for data mining where researchers can discover a tremendous amount of information hidden in the literature [92, 93]. The majority of studies adopting the data mining approach use text mining techniques.

#### Text mining

Text mining as applied to the drug repositioning problem is typically used to find data related to a particular gene, disease, or drug specified and then classify the relevant entities or knowledge from the retrieved data based on the co-occurrence between the relevant entities or by using natural language processing. For instance, if drug *R* is connected with gene *G*, and gene *G* is related to disease *D*, then drug *R* may have a new connection with disease *D*. Generally, text mining includes four steps which are: (1) Information retrieval (IR), (2) Entity recognition (NER), (3) Information extraction (IE), and (4) Knowledge discovery (KD) [94].

Cheng et al. [95] developed a web-based text mining system for extracting relationships between different biological terms such as diseases, tissues, genes, proteins, and drugs by using a variety of text mining and information retrieval techniques over a massive set of existing biological databases in order to identify, highlight and rank informative abstracts, paragraphs or sentences. Li and Lu [96] introduced a model to identify clinical pharmacogenomics (PGx) gene-drug-disease relationships from clinical trial data. The authors determined text of interest in clinical trial records retrieved from ClinicalTrials.gov [77] and used a dictionary to identify PGx concepts. Then, they considered the co-occurrence of PGx concepts in each clinical trial to define gene-drug-disease relationships. Finally, they indexed each clinical trial using its identified gene-drug-disease relationships. Therefore, given a PGx gene, the introduced model can identify related diseases and drugs within the corresponding clinical trials. Likewise, given a pair of PGx gene-drug or gene-disease, the introduced model can return clinical trials in which the PGx pair is or has been studied.

Leaman et al. [97] built a tool for recognizing disease entities mentioned in literature. The authors used disease corpus from the National Center for Biotechnology Information (NCBI) [46] and the MEDIC vocabulary [98] to single out diseases mentioned in PubMed abstracts and subsequently handle abbreviations. Afterward, they used pairwise learning to rank, which has proven to be successful in information retrieval, for normalizing mentioned text and identifying MEDIC concepts for the disease entities mentioned in PubMed abstracts.

Text mining has also been widely used successfully for discovering relationships between genes, diseases, and drug [99], investigating gene-gene interactions [100], and building a heterogeneous network of genes, diseases, and drugs [27]. Li et al. [101] proposed a new approach that integrates literature text-mining data with protein interaction networks to build a drug-protein connectivity map for a specific disease. The authors used Alzheimer’s disease (AD) as a case study and showed that their approach outperformed curated drug-target databases and conventional information retrieval systems and also suggested two existing drugs as candidate drugs for AD treatment.

Unlike common text mining approaches where biological networks are built based on the co-occurrence of biological entities, Tari et al. [102] introduced a novel approach that considered interaction types, interaction type directions, and drug mechanism representation. The authors used text mining to obtain data from publicly available sources that then used to produce a set of logical facts. Then, the set of logical facts was used along with logical rules that represent drug mechanism properties to build an automated reasoning model for identifying therapeutic potentials and novel indications for existing drugs. Rastegar-Mojarad et al. [103] used text mined data in order to identify drug-gene and gene-disease semantic predictions, which then were utilized to compile a list of potential drug-disease pairs. Finally, the authors ranked the drug-disease pairs using the predicates between drug-gene and gene-disease pairs, evaluated their model against two different datasets, and concluded that the combination of drug-gene and gene-disease predicates could eventually be used to highlight the drugs in the top-ranked drug-disease pairs as drug repositioning candidates.

Brown et al. [104] proposed a web-based text mining system for drug repositioning. The authors used the number of shared indications across drug–drug pairs to disclose similarity among these drug–drug pairs and then clustered drugs based on their similarity, which revealed both known and novel drug indications. Papanikolaou et al. [105] applied text mining on the DrugBank database’s text attributes to identify drug–drug associations. The authors used Name Entity Recognition (NER) to identify biological entities (proteins, genes, diseases, etc.) in the DrugBank’s description, indication, pharmacodynamics, and mode-of-action text fields. Then, they used an algorithm to eliminate any insignificant terms and created a binary vector representing each DrugBank record. Finally, they clustered DrugBank records using several clustering algorithms and similarity measures. Such an approach can facilitate the retrieval of novel drug–drug associations, which may significantly contribute to new drug repositioning applications.

Recently, Zeng et al. [106] introduced a deep-learning approach where they retrieved data from various publicly available sources to build ten heterogeneous networks to identify potential drug-disease associations. The proposed approach outperformed conventional approaches in discovering novel drug-disease associations when its findings were examined using cross-validation, external validation, and case studies. Moreover, the approach suggested several potential drug repositioning candidates for Alzheimer’s and Parkinson’s disseases. Han et al. [99] leveraged text mining of OMIM phenotypes to construct a phenotype network and used Graph Convolutional neural Network (GCNN) to identify disease-gene interactions by focusing on non-linear disease-gene correlations. The authors found out that their approach surpassed all other state-of-the-art methods on the majority of metrics.

#### Semantic technologies

Semantic technologies have allowed to easily combine data from different sources to predict therapeutic potentials and novel indications for existing drugs. For example, Chen et al. [107] proposed a statistical model based on the network’s topology and semantics of the sub-network between a drug and a target to predict drug-target associations in a linked heterogeneous network composed, semantically, of annotated data obtained from various publicly available sources, including protein-protein, drug–drug, and drug-side effects, etc. The model successfully differentiated between already known direct drug-target associations and random drug-target associations with high accuracy and identified indirect drug-target associations. Moreover, a drug similarity network signalled that drugs with very different indications from different disease areas are clustered with each other, which may suggest therapeutic potentials and new indications for these drugs.

Zhu et al. [108] used clinical pharmacogenomics (PGx) data, including relations among drugs, diseases, genes, pathways, and single nucleotide polymorphisms (SNPs), and Semantic Web to generate pharmacogenomics Web Ontology Language (WOL) profiles and identify pharmacogenomics associations for FDA approved breast cancer drugs. The authors evaluated their approach using several case studies and indicated that leveraging semantic web technology while studying pharmacogenomics data could lead to higher standard findings of novel drug-disease associations and drug indications.

### Machine learning

Computational drug repositioning has evolved over the past two decades from naïve drug similarity attempts, which often used a single source of biological or biomedical data, into an innovative application domain for machine learning approaches. Similar to machine learning models in other domains, computational drug repositioning models require an extensive amount of data to train these models and come up with robust decision rules, aiming to reveal the underlying associations between biological and biomedical entities. The tremendous growth in the volume of publicly available biological and biomedical data and the valuable advancement resulting from machine learning models in other disciplines has assisted the considerable effort in the creation, study, and use of machine learning methods for discovering novel drug-disease associations and drug repositioning applications. Such methods used Naïve Bayesian, k-nearest neighbors (kNN) [109], random forest [110], support vector machines (SVM) [111], and more recently deep neural networks [112] for binary classification, multiclass classification, and values prediction.

#### Classification

Gottlieb et al. [113] leveraged various data sources to predict drug-disease associations. The authors used drug–drug (e.g., chemical structure, side effects, etc.) and disease–disease (e.g., gene expressions, phenotype, etc.) similarity measures as classification features. Then, they applied a logistic regression classifier to distinguish between true and false drug-disease associations and eventually predict novel drug-disease associations.

Menden et al. [114] developed machine learning models to predict the reactions of cancer to drug treatment using the combination of cell lines genomics and drug chemical structures. The authors integrated both data sources to build a feed-forward perceptron neural network model and a random forest regression model and then validated their findings by cross-validation and an independent blind test. They claimed that the utilization of such models could go further than virtual drug screening since it systematically tested drug efficiency and thus identified potential drug repositioning applications and ultimately could be useful for personalized medicine by linking the cell lines genomics to drug intolerance.

#### Collaborative filtering

It is noteworthy that several studies based on machine learning have applied collaborative filtering, which depends on historical trends such as gene expression in different samples, to predict novel drug indications and drug-disease associations. Napolitano et al. [115] used several drug-related similarity datasets as feathers to predict the therapeutic class of FDA-approved compounds and intentionally considered any mismatches between known and predicted drug classifications as potential alternative therapeutic indications. The authors combined three drug–drug similarity datasets, based on gene expression signatures, chemical structures, and molecular targets, into one drug similarity matrix, which was used as a kernel to train a multi-class Support Vector Machine (SVM) classifier. Afterward, they utilized collaborative filtering techniques to predict novel drug-disease indications.

Zhang et al. [116] introduced a unified computational framework for integrating numerous biological and biomedical sources in order to infer novel drug–drug similarities as well as disease–disease similarities. The authors incorporated drug similarities (e.g., target proteins, side effects, and chemical structure), disease similarities (e.g., gene-disease associations and disease phenotype), and known drug-disease associations datasets to build a drug-disease network. The drug-disease network was treated as an optimization problem, which was solved using block coordinate descent (BCD) strategy. The results demonstrated that such a framework could be useful in finding novel drug-disease associations and identifying new drug repositioning opportunities.

Yang et al. [117] presented a causal inference-probabilistic matrix factorization (CI-PMF) approach to identify and classify drug-disease associations. The authors used several biological and biomedical sources (e.g., drug targets, pathways, pathway-related genes, and disease-gene associations) to build a causal network that linking drug, target, pathway, gene, disease entities together in order to rank drug-disease associations. Furthermore, they leveraged known drug-disease associations to form a probabilistic matrix factorization (PMF) model, which was used to construct a PMF model to classify constructed drug-disease associations into different classes. Finally, they exploited drug-disease association ranking scores and predicted classes to identify novel drug-disease association.

Lim et al. [118] conducted a large-scale study to infer off-target drug interactions and identify novel drug repositioning candidates. The authors used drug chemical structures and protein targets data to build a dual regularized one-class collaborative filtering model that surpassed the previously introduced state-of-the-art models. Ozsoy et al. [119] treated the drug repositioning process as a recommendation process and utilized Pareto dominance and collaborative filtering to identify drug-disease associations. The authors integrated multisource drugs data (protein targets, chemical structures, and side effects) and applied a variety of similarity measures to calculate drug–drug similarities and then used a Pareto dominance model to identify neighbor drugs. Finally, they used diseases that are shared among neighbor drugs to infer potentials and novel indications for existing drugs.

#### Deep learning

With the significant growth in publicly available datasets and rapid increase in computational power, deep learning (DL), or neural network (NN), has gained considerable attention. As an inspiring machine learning division, deep learning has given a significant boost and emerged as the leading technique for drug discovery and development in the most recent published studies [112, 120].

Deep learning, a notion closely linked to artificial neural networks (ANNs), can be defined as the learning from nonlinear processing of interconnected neurons layers. It has attracted researchers for its architecture’s flexibility, which enables the development of single task or multitask machine learning models for identifying potential therapeutic applications and predicting drug-disease interactions. Although deep learning has been utilized to develop up-and-coming models in the drug repositioning field, it is worth emphasizing that the full-power employment of deep learning still has some limitations. For instance, deep neural network models need to be adjusted to fit the data used in training these models, which takes substantial time and effort. Additionally, the selection of which machine learning technique or similarity measure to use with each dataset in the deep neural network layers is not straightforward and somehow depending on the used datasets. Neural networks can be mainly classified, based on network’s architecture, into (1) fully-connected deep neural network (DNN), (2) convolutional neural network (CNN), (3) recurrent neural network (RNN), (4) autoencoder (AE) [112].

Aliper et al. [121] employed a fully-connected deep neural network to predict the pharmacological properties of drugs and identifying therapeutic potentials and novel drug indications. The authors used gene expression signatures data and pathways data to build deep neural networks models which outperformed support vector machine model and achieved high classification accuracy in predicting drug indications and, hence such deep neural networks could be useful for drug repurposing. Furthermore, they proposed using deep neural net confusion matrices for drug repositioning.

Altae-Tran et al. [122] integrated a standard one-shot learning paradigm with a convolutional neural network to come up with an iterative refinement long short-term memory (LSTM) learning model. The authors adopted the standard one-shot learning paradigm to enhance the learning of meaningful distance metrics over small-molecules in new experiment systems. When evaluated against two different related datasets, the proposed one-shot model achieved remarkable success in identifying molecular behaviour in low-data drug discovery experiments.

Hu et al. [123] introduced a convolutional neural network model to unveil drug-target interactions. The authors used drug chemical structures and protein sequences data to construct their convolutional neural network classifier that showed superior performance in comparison with other state-of-the-art models. The proposed model inferred drug-target associations in the case of having multiple target proteins interacting with multiple chemical molecules, which demonstrate the potential of such a model in identifying therapeutic novel indications and drug repositioning opportunities.

Segler et al. [124] proposed a recurrent neural network model to generate novel molecules for drug repositioning applications. The authors used drug structures and drug-target interactions data to train their recurrent neural network classifier to produce new molecules that are strongly associated with the desired biological targets. The proposed model was evaluated against two different known drug-target association datasets and performed fairly well. However, the introduced model mimicked the complete de novo drug design cycle and generated large sets of novel molecules when it was integrated with a scoring function.

Zeng et al. [106] used multi-modal deep autoencoder and variational autoencoder models to discover drug-disease associations. The authors integrated various drug-related datasets (drug-disease associations, drug-target associations, drug–drug associations, and drug side effects) to train a multi-modal deep autoencoder and then define high-level drug features. After that, they encoded and decoded the combination of high-level drug features and clinically reported drug-disease associations using variational autoencoder to identify novel therapeutic indications in addition to already identified indications. The findings were validated against a well-known dataset of drug-disease associations and surpassed the previous state-of-the-art machine learning models. Furthermore, the authors reported drug repositioning candidates for Alzheimer’s and Parkinson’s diseases.

### Network analysis

Networks and their analysis have been excessively used in the field of computational drug repositioning as they can provide considerable insight into drug mode-of-action and indications and how drug targets work and, therefore, identify therapeutic potentials and unveil drug repositioning applications. Networks are an excellent way of modelling biological and biomedical entities and their interactions and relationships. Such models can, in turn, be used to discover informative relationships by leveraging graph theory concepts, statistical analysis, and computational models. In such networks, nodes are used to represent genes, proteins, molecules, phenotypes, or any other biological or biomedical entities, and edges are used to represent functional similarities, mode-of-actions, underlying mechanisms, or any other relationships. Additionally, nodes and edges can be weighted to represent specific attributable information. Moreover, integrating different entities/relationships in a network result in a heterogeneous network while focusing on one entity class or relationship produces a homogeneous network.

Like other computational drug repositioning approaches, drug-based strategy studies, as well as disease-based studies, have also benefited from the network analysis approach to infer drug-target associations and identify novel drug repositioning candidates. Studies based on network analysis can be categorized, according to their data sources, into categories: gene regulatory networks, metabolic networks, protein-protein interaction networks, drug-target interaction networks, drug–drug interaction networks, drug-disease association network, drug-side effect association networks, disease–disease interaction networks, and integrated heterogeneous networks.

#### Bipartite graph

Yamanishi et al. [30] proposed a bipartite graph supervised learning model to infer novel drug-target interactions. The authors combined protein-protein interaction information with drug chemical structure information and drug-target interaction network to predict different drug-target interaction classes, which could significantly help in improving drug repositioning research productivity. Kinnings et al. [31] built a drug–drug interaction network to unveil drug communities within the network and eventually identify therapeutic potentials and novel indications for existing drugs. The authors represented drugs as nodes and used drug chemical structure information and drug-target interactions similarity to draw edges between drugs. Afterward, they studied the drug–drug interaction network and came up with drug repositioning candidates that were validated using case studies.

Hu and Agarwal [13] constructed a disease-drug network to identify drug repositioning applications and discover drug side effects. The authors used microarray gene expression profiles to build a disease-drug network, which they then enriched using CMap data. The proposed model was validated against gold-standard data and showed high potential in identifying novel therapeutic indications for existing drugs. Li and Lu [35] develop a novel bipartite graph model to infer drug-target indications based on drug pairwise similarity. The authors used drug chemical structure information along with drug-targets interactions information to build their supervised learning bipartite graph model, which captured the implicit information between drug targets and surpassed other state-of-the-art models.

#### Clustering

Wu et al. [125] built a weighted drug-disease heterogeneous network and applied network clustering to identify potential drug repositioning candidates within closely connected network modules. The authors used disease-gene associations and drug-target interactions to construct their weighted heterogeneous network where drugs and diseases were defined as nodes, edges were drawn when a pair of nodes share genes, targets, biological processes, pathways, phenotypes, or a combination of these features, and edges were weighted using Jaccard coefficient similarity. Subsequently, they used two network clustering algorithms to cluster nodes into modules and then assembled all potential drug-disease pairs within each of these modules. Finally, they treated drug-disease pairs suggested by the two network clustering algorithms as drug repositioning candidates and performed literature validations and presented several case studies in support of their proposed model.

Tan et al. [38] built a drug–drug interaction network in order to identify novel drug target indications. The authors utilized drug chemical structure information, gene semantic similarity information, and drug-target interaction networks to calculate the degree of drug similarity which then used to construct a drug–drug interaction network, neighbor drugs by clustering the drug–drug interaction network into modules based on mode-of-action, and finally propose new drug therapeutic indications. The proposed model showed high accuracy when validated using the literature.

#### Network centrality measures

Rakshit et al. [126] developed a novel network-based bidirectional top-down and bottom-up approaches to predict potential drug repositioning applications for a specific disease. The authors used disease-specific (Parkinson’s disease) target information and drug-target indications to construct two networks. Subsequently, they utilized several network centrality measures to identify genes and drugs of interests in both networks and used them as an input for the top-down and bottom-up models. The introduced models identified a set of drug repositioning candidates to be investigated for Parkinson’s disease treatment, which was validated against a well-known drug-target indications data source.

Yang et al. [127] proposed a new systematic model to identify therapeutic potentials and drug repositioning candidates in heterogeneous networks. The authors combined molecular data, side effects, and online health community information to construct a heterogeneous network that consists of drugs, diseases, and adverse drug reactions as intermediates. Subsequently, they applied several path-based heterogeneous network mining models to identifying and drug repositioning candidates and literately validated their models and concluded that the more data sources used for constructing such heterogeneous networks, the better for predicting models.

## Validation of computational drug repositioning models

Ideally, computational drug repositioning studies are conducted to identify new uses for already existing drugs and optimize the pre-clinical process of developing new drugs by saving time and cost compared to the traditional de novo drug discovery and development approach. Researchers validate/evaluate their findings and conclude their models by recommending a set of drug repositioning candidates.

**Table 2:** An overview of computational drug repositioning studies, their adopted strategies, computational approaches, main techniques, data sources, key findings, and evaluation metrics

Study	Strateg(ies)			Computational approach(es)			Main technique(s)	Data source(s)	Evaluation criteria	Key finding(s)
	Genome	Chemical structures	Phenome	Data mining	Machine learning	Network analysis				
Hu et al. [123]	$$\checkmark$$	$$\checkmark$$			$$\checkmark$$		CNN	KEGG and DrugBank	CV, ROC and Comp(Acc, Sen, F1, and AUC)	R-TIs
Han et al. [99]	$$\checkmark$$		$$\checkmark$$	$$\checkmark$$	$$\checkmark$$		TM, GCN, and MF	OMIM	CV and Comp(MAP, AUC, and NDCG)	D-GAs
Zeng et al. [106]	$$\checkmark$$		$$\checkmark$$		$$\checkmark$$		MM-AE and V-AE	DrugBank, repoDB, and ClinicalTrials.gov	CV, AUC, AUC-PR, and Comp(Rec-TK)	R-DIs
Yang et al. [127]	$$\checkmark$$		$$\checkmark$$			$$\checkmark$$	ARM and LP	PharmGKB, SIDER, and MedHelp [128]	CV, AUC, AUC-PR, and Comp(Send, Spc, and F1)	R-DIs
Ozsoy et al. [119]	$$\checkmark$$	$$\checkmark$$	$$\checkmark$$		$$\checkmark$$		SM, PDo, and CF	PubChem, UniProt, SIDER, and TIRs [35]	CV and Comp(Prec, Rec, F1, Spc, and AUC)	R-DIs
Segler et al. [124]		$$\checkmark$$			$$\checkmark$$		RNN	SMILES	EOR	R-DIs
Altae-Tran et al. [122]		$$\checkmark$$	$$\checkmark$$		$$\checkmark$$		CCN and LSTM	SIDER and Tox21 [129]	Acc and AUC	R-DIs
Papanikolaou et al. [105]		$$\checkmark$$		$$\checkmark$$			NER	DrugBank	—	R-RAs
Brown et al. [104]		$$\checkmark$$		$$\checkmark$$	$$\checkmark$$		SM and Clust	MEDLINE and DrugBank	CSs	R-DIs
Nugent et al. [59]			$$\checkmark$$	$$\checkmark$$			SCM	Twitter	Comp(Acc)	R-DIs
Aliper et al. [121]	$$\checkmark$$				$$\checkmark$$		DNN	LINCS	CV, F1, NNCM, Spr, and Comp(Acc)	R-RAs
Lim et al. [118]	$$\checkmark$$	$$\checkmark$$			$$\checkmark$$		SM and CF	ZINC [130]	CV and Comp(Acc,and Rec)	R-TIs
Sridhar et al. [60]	$$\checkmark$$	$$\checkmark$$	$$\checkmark$$		$$\checkmark$$		SM and PSL	DrugBank	Comp(AUC, AUC-PR, F1) and CSs	R-RIs
Zheng et al. [39]		$$\checkmark$$		$$\checkmark$$	$$\checkmark$$		SM	BindingDB [131]	CV, Acc, Spc, Sen, AUC, Prec, F1, Comp(AUC, Sen, and Spc), and CSs	R-TIs
Rastegar-Mojarad et al. [103]	$$\checkmark$$			$$\checkmark$$		$$\checkmark$$	TM and SM	SemMedDB [132], UMLS [133], CTD [134], and Medline	Comp(CoS)	R-DIs
Rakshit et al. [126]	$$\checkmark$$			$$\checkmark$$		$$\checkmark$$	TM and Genotator [135], PolySearch [95], Pescador [136], and DrugBank	Comp(Acc)	R-TIs	
Huang et al. [27]	$$\checkmark$$				$$\checkmark$$	$$\checkmark$$	SM and BGL	CMap	CV, AUC, Comp(AUC), and CSs	R-TIs
Zhu et al. [108]	$$\checkmark$$	$$\checkmark$$		$$\checkmark$$			SM and WOL	PharmGKB	CSs	R-DIs
Tari et al. [102]	$$\checkmark$$	$$\checkmark$$		$$\checkmark$$			TM and LFRs	Medline, GO, UniProt, NCBI, CancerQuest [137], and DrugBank	Comp(Rec) and CSs	R-DIs
Okada et al. [23]	$$\checkmark$$	$$\checkmark$$		$$\checkmark$$			GWAS	GWASs	P and CSs	R-DIs
Yang et al. [117]	$$\checkmark$$	$$\checkmark$$			$$\checkmark$$		CI-PMF	DrugBank, BioCarta [138], and CTD [134]	Comp(AUC and Prec) and CSs	R-DIs
Zhang et al. [116]	$$\checkmark$$	$$\checkmark$$	$$\checkmark$$		$$\checkmark$$		SM and BCD	DrugBank, NDF-RT [139], and HPRD [140]	CV, Comp(AUC), and CSs	R-DIs
Bisgin et al. [58]			$$\checkmark$$		$$\checkmark$$	$$\checkmark$$	SM and LDA	SIDER	Acc and CSs	R-TIs
Tan et al. [38]	$$\checkmark$$	$$\checkmark$$				$$\checkmark$$	SM and Clust	PubChem and DrugBank	Comp(Acc)	R-TIs
Ye et al. [57]			$$\checkmark$$			$$\checkmark$$	SM	SIDER	CV, Acc, and CSs	R-TIs
Menden et al. [114]	$$\checkmark$$	$$\checkmark$$			$$\checkmark$$		FF-PNN and RF	CCL [18]	CV, Acc, $$R_{p}$$, $$R^{2}$$, RMSE, and CSs	R-TIs
Napolitano et al. [115]	$$\checkmark$$	$$\checkmark$$			$$\checkmark$$		SVM and CF	CMap, NCBI, DrugBank, and ATC [141]	CV, Acc, Comp(AUC)	R-DIs
Wang et al. [37]	$$\checkmark$$	$$\checkmark$$	$$\checkmark$$		$$\checkmark$$	$$\checkmark$$	SM, KF, and SVM	PubChem, KEGG, BRENDA [142], SuperTarget [143], DrugBank, and SIDER	CV, Comp(AUC, Acc, Sens, Spc, Prec, and F1), $ CSs	R-DIs
Gottlieb et al. [56]	$$\checkmark$$	$$\checkmark$$	$$\checkmark$$	$$\checkmark$$	$$\checkmark$$		SM	DrugBank, Drugs.com, and SIDER	CV, Acc, AUC, and CSs	R-TIs
Wu et al. [125]	$$\checkmark$$	$$\checkmark$$	$$\checkmark$$		$$\checkmark$$	$$\checkmark$$	SM and Clust	KEGG	CV, Comp(Acc), and CSs	R-DIs
Chen et al. [107]	$$\checkmark$$		$$\checkmark$$		$$\checkmark$$	$$\checkmark$$	SM and LP	Chem2Bio2RDF [144], DrugBank, and Matador [143]	Acc, AUC, Copm(Acc and AUC), and CSs	R-TIs
Li and Lu [96]	$$\checkmark$$			$$\checkmark$$		$$\checkmark$$	TM	ClinicalTrials.gov and PharmGKB	Acc, Comp(Acc), and CSs	PGx
Li and Lu [35]	$$\checkmark$$	$$\checkmark$$	$$\checkmark$$	$$\checkmark$$		$$\checkmark$$	SM and BGL	DrugBank, NDF-RT [139], HPRD [140], and ClinicalTrials.gov	CV (Sen, Spc, and AUC), Comp(Spc, Sen, and AUC), and CSs	R-TIs
Hoehndorf et al. [55]	$$\checkmark$$		$$\checkmark$$		$$\checkmark$$		SM	PhenomeNET [145] and PharmGKB	AUC and CSs	R-TIs
Gottlieb et al. [113]	$$\checkmark$$	$$\checkmark$$	$$\checkmark$$		$$\checkmark$$		SM and LR	DrugBank, SMILES, Matador [143], DCDB [146], KEGG, DailyMed [147], SIDER, GO, UniPort, and ClinicalTrials.gov	CV, Acc, P, AUC, and Comp(Acc and AUC)	R-DIs
Sirota et al. [14]	$$\checkmark$$				$$\checkmark$$		Clust and SM	GEO and CMap	CSs	R-DIs
Hu and Agarwal [13]	$$\checkmark$$					$$\checkmark$$	SM	GEO, CMap, and DrugBank	Acc, Comp(Rec and Sens), and CSs	R-DIs
Li et al. [101]	$$\checkmark$$	$$\checkmark$$		$$\checkmark$$	$$\checkmark$$		TM, SM and CMap	OPHID [148], PubMed	CV, Comp(Sen, Spc, PPV, F1, and Acc), and CSs	R-DIs
Bleakley et al. [32]	$$\checkmark$$	$$\checkmark$$			$$\checkmark$$	$$\checkmark$$	SM, BGL, and SVM	KEGG, BRENDA [142], SuperTarget [143], and DrugBank	CV, Comp(AYC and AUC-PR), and CSs	R-TIs
Kinnings et al. [31]	$$\checkmark$$	$$\checkmark$$				$$\checkmark$$	SM	MDDR [149]	Comp(Acc) and CSs	R-TIs
Keiser et al. [9]		$$\checkmark$$			$$\checkmark$$	$$\checkmark$$	SM and BGL	KEGG, BRENDA [142], SuperTarget [143], and DrugBank	CV, Comp(AUC, Sens, Spc, and PPV), and CSs	R-TIs
Yamanishi et al. [30]	$$\checkmark$$	$$\checkmark$$			$$\checkmark$$	$$\checkmark$$	SM and BGL	KEGG, BRENDA [142], SuperTarget [143], and DrugBank	CV, Comp(AUC, Sens, Spc, and PPV), and CSs	R-TIs
Cheng et al. [95]	$$\checkmark$$			$$\checkmark$$		$$\checkmark$$	TM	PubMed, UniPort, HGMD [150], DrugBank, HMDB [151]	Comp(F1) and CSs	BER
Campillos et al. [53]	$$\checkmark$$	$$\checkmark$$	$$\checkmark$$		$$\checkmark$$		SM	Matador [143], DrugBank, PDSP Ki [152], and STRING [153]	Comp(Spc and Sen) and CSs	R-DIs
Lamb et al. [12]	$$\checkmark$$				$$\checkmark$$		Clust and CMap	CHC	CSs	R-DIs

*Acc* Accuracy, *AE* Autoencoder, *ARM* Association Rules Mining, *AUC* Area under curve, *AUC-PR* Area Under The Precision-Recall Curve, *BCD* Block Coordinate Descent, *BER* Biological Entity Relationships, *BGL* Bipartite Graph Learning, *CF* Collaborative Filtering, *CHC* Cultured Human Cells, *CI-PMF* Causal Inference-Probabilistic Matrix Factorization , *Clust* Clustering, *CMap* Connectivity Map , *CNN* Convolutional Neural Network , *Comp* Comparison With Other Approaches, *CoS* Co-occurrence Score, *CSs* Case Studies, *CV* Cross Validation , *D-GAs* Disease-Gene Associations, *DNN* Deep Neural Network, *EOR* Enrichment Over Random, *F1* F1 score/F-measure, *FF-PNN* Feed-Forward Perceptron Neural Network, *GCN* Graph Convolutional Network, *GWAS* Genome-Wide Association Study, *KF* Kernel Fusion, *LDA* Latent Dirichlet Allocation, *LFRs* Logical Facts and Rules, *LP* Link Prediction, *LR* Logistic Regression, *LSTM* Long Short-Term Memory, *MAP* Mean Average Precision, *MF* Matrix Factorization, *MM-AE* Multi-modal Deep Autoencoder, *NDCG* Normalized Discounted Cumulative Gain, *NER* Name Entity Recognition, *NNCM* Neural Net Confusion Matrix, *P* P-value, *PDo* Pareto Dominance, *PGx* Pharmacogenomics, *PPV* Positive Predictive Value, *Prec* Precision, *PSL* Probabilistic Soft Logic, ‘$R^{2}$’: Coefficient of Determination , ‘$R_{p}$’: Pearson Correlation, *R-DIs* Drug-Disease Interactions, *Rec* Recall, *Rec-TK* Recall @ Top-K, *RF* Random Forest, *RMSE* Root Mean Square Error, *RNN* Recurrent Neural Network, *ROC* Receiver Operating Characteristic Curve, *R-RAs* Drug–Drug Associations, *R-TIs* Drug-Target Interactions, *SCM* Sample Covariance Matrix, *Sen* Sensitivity, *SM* Similarity Measures, *Spc* Specificity, *Spr* Separability, *SVM* Support Vector Machine, *TIRs* Therapeutic Indication Relationships, *TM* Text Mining, *V-AE* Variational Autoencoder, *WOL* Web Ontology Language

However, validation/evaluation models might differ, in contexts, from the proposed computational models, or specific validation models might not be accurate and trustworthy. Thus, comprehending and picking out suitable validation models is highly crucial for the success of the proposed computational models. Furthermore, selecting the right set of drug repositioning candidates for validation is crucial too due to different factors, such as high price, high level of toxicity, and reduced bioavailability, and due to certain drugs having been abandoned or not preferred by physicians or biologists. Therefore, it is essential that all interested parties are deeply engaged in the process of drug repositioning to boost the conducted research in this field.

Practically speaking, validation/evaluation models vary from one study to another and can depend, up to a certain extent, on the nature of desired outcomes. These models can be classified into (1) in vitro experiments (2) in vivo experiments (3) electronic health records (4) leave-one-out and cross-validation (5) benchmarking against previous models (6) case studies (7) literature cross-referencing, and (8) domain experts consultation.

Despite some well-known drawbacks, in vitro and in vivo experimental validation models have been widely used to validate drug repositioning candidates. In vitro and in vivo validation models refer to performing experiments in a controlled environment outside of a living organism (e.g., cellular biology studies outside of organisms or cells) and in a whole living body (e.g., animal studies and clinical trials) respectively. For example, Lim et al. [118] identified albendazole as a drug repositioning candidate for anti-cancer effects and presented in vitro and in vivo pieces of evidence in support of using it to treat liver cancer and ovarian cancer.

In order to evaluate the efficiency of potential repositioned drugs, Rakshit et al. [126] introduced a metric called On-Target Ratio (OTR) which is the ratio between the number of drug targets in their proposed disease-specific genes network to the total number of interactions of the same drug in the DrugBank database. Moreover, Ozsoy et al. [119] evaluated their results against ClinicalTrials.gov, which is a collection of publicly and privately funded clinical studies from around the world. The authors also performed a leave-one-out test and benchmarked their model against state-of-the-art models.

Yang et al. [127] used scientific articles published by PubMed as a medical literature cross-referencing model to evaluate the performance of their proposed models. Furthermore, the authors consulted medical experts to evaluate their findings and guarantee the accuracy of their proposed model. The medical experts indicated that the repositioning drugs candidates identified by the proposed model offered significant benefit in filtering and reducing the number of drugs that can be possibly used for the suggested indications. In addition to using an electronic health records validation model, Zeng et al. [106] presented two case studies to validate their proposed deep learning model, which identifies potential drug-disease associations. The authors used Alzheimer’s disease and Parkinson’s disease to showcases how robust their proposed model is and suggested approved drugs for Alzheimer’s disease (e.g., risperidone and aripiprazole) and Parkinson’s disease (e.g., methylphenidate and pergolide).

It is noteworthy that literature-based validation models have been wildly adopted in recent studies as literature mining approaches have snowballed. Additionally, K-fold cross-validation is often used to train models in machine learning-based studies to overcome the over-optimistic estimation of model performance, which can also be tackled using a new testing dataset independent of the training set, assuming that such information is available.

**Table 3:** Examples of drug repositioning applications in various disease areas and related therapeutics

Disease area	Drug name/active ingredients	Original indication	New indication(s)	New indication(s) status
Depression	Duloxetine hydrochloride	Major Depressive Disorder (MDD)	Neuropathic pain, generalized anxiety disorder (GAD), osteoarthritis, and stress incontinence	Approved
	Fluoxetine hydrochloride	Major Depressive Disorder (MDD)	Premenstrual dysphoric disorder (PMDD)	Approved
	Sibutramine hydrochloride	Major Depressive Disorder (MDD)	Obesity	Approved
Neurology	Atomoxetine hydrochloride	Parkinson’s disease (PD)	Attention-deficit hyperactivity disorder (ADHD)	Approved
	Ropinirole hydrochloride	Hypertension (HTN)	Parkinson’s disease (PD)	Approved
Non-neurology	Minoxidil	Hypertension (HTN)	Hair loss	Approved
	Finasteride	Benign prostatic hyperplasia (BPH)	Hair loss	Approved
	Zidovudine	Failed clinical trials for cancer	Human immunodeficiency virus (HIV)	Approved
	Sildenafil	Angina	Erectile dysfunction (ED) and pulmonary arterial hypertension (PAH)	Approved
Cancer	Auranofin	Rheumatoid arthritis (RA)	Gastrointestinal stromal tumor (GIST)	Approved
	Imatinib	Chronic myeloid leukemia (CML)	Gastrointestinal stromal tumors (GIST)	Approved
	Irinotecan hydrochloride	Colorectal cancer	Pancreatic cancer	Approved
	Nelfinavir	Human immunodeficiency virus 1 (HIV-1)	Colorectal cancer, lung cancer, cervical cancer, pancreatic cancer, ovarian cancer, metastatic cancer	Investigational
	Metformin hydrochloride	Type 2 diabetes (T2DM)	Breast cancer, pancreatic cancer, endometrial cancer, colorectal cancer, and esophageal cancer	Investigational
	Trastuzumab	Human epidermal growth factor receptor 2 (HER2)-positive breast cancer	Metastatic breast cancer, gastric cancer, and early breast cancer	Approved
	Sunitinib	Renal cell carcinoma (RCC) and Gastrointestinal stromal tumor (GIST)	Pancreatic neuroendocrine tumors (PNETs)	Approved
	Crizotinib	Clinical trials for anaplastic large cell lymphoma (ALCL)	Non-small cell lung cancer (NSCLC)	Approved
Infectious	Thalidomide	Morning sickness (withdrawn)	Erythema nodosum leprosum (Leprosy)	Approved
	Everolimus	Immunosuppressant	Pancreatic neuroendocrine tumors (PNETs), renal cell carcinoma (RCC), and subependymal giant cell astrocytoma (SEGA)	Approved
	Sirolimus	Organ rejection in patients receiving renal transplants	Malaria	Investigational
Rare and orphan	Alefacept	Chronic plaque psoriasis	Rejection in patients receiving allogeneic solid organ transplants	Investigational
	Indium In-111 pentetreotide	Agent for the scintigraphic localization of primary and metastatic neuroendocrine tumors	Neuroendocrine tumors (NETs)	Investigational

## Current and prospective drug repositioning applications

As a result of reviewing a number of computational drug repositioning studies and zooming in into their findings, we have identified a set of disease areas and related therapeutics that have benefited from drug repositioning applications. When drug repositioning started to get the scientific community attention, a number of studies were conducted to learn about mode-of-action for antidepression, neurological, and non-neurological drugs. These studies have successfully unveiled new indications for already approved drugs as well as drugs in the pipeline.

In Table 3, a number of successes are listed with their original indication as well as the new and in most of the cases approved indication. There are five drugs with the original indication being for aspects of the nervous system (depression and neurology). The new indications are also for aspects of the nervous system. The new indications included a new medicine to treat obesity, a disease of plenty. As reported by the World Health Organization in 2020, 650 million people are obese worldwide [154]. The new indication for treating obesity is, therefore, a significant step forwards.

Cancer is another area where a number of new drug indications have been found. Cancer is also a disease that is on the rise. Globally an estimated 10 million people die of cancer each year [155], and many more continue a reduced lifestyle as they are combatting the effects of cancer. Furthermore, the incidence of this disease increases with age. Since the average age of populations is on the rise, it is expected that this will lead to a higher number of people with cancer. The new drug indications for various cancers are, therefore, of the highest importance. Pessetto et al. [156] conducted a high-throughput screening study on FDA-approved drugs and found that *auranofin* could be repositioned for the treatment of gastrointestinal stromal tumours. Stenvang et al. [157] applied a biomarker-guided repurposing approach on genome information and clinical studies and proposed *irinotecan* for the treatment of breast cancer.

Infectious diseases are caused by pathogenic microorganisms such as bacteria and viruses. Multi-drug resistance and extensively antibiotic drug-resistant microbes threaten the treatment of such diseases and require new processes of treatment. Drug repositioning has led to success in combating infectious diseases. Ng et al. [158] proposed an integrated chemical genomics and structural systems biology approach which identified *plasmodium falciparum* targets of drug-like active compounds from the malaria box, and suggested that several approved drugs may be active against malaria.

Rare and orphan diseases affect a small proportion of the world ’s population. A key motivator behind developing a treatment for an incurable disease is the potential market size for the treatment. As a result, thousands of rare and orphan diseases lack treatments because of the insignificant potential market size for these treatments. Drug repositioning has gained some attention in identifying therapeutics for orphan and rare diseases. Molineris et al. [159] utilized several resources (e.g., OMIM, DrugBank, CMap) to conduct a systematic analysis of gene co-expression and successfully identified HDAC1 and TSPO as two significant targets for epileptic syndromes. Xu et al. [160] used FDA orphan designation database and FDA-approved drugs to establish the Rare Disease Repurposing Database (RDRD). RDRD provides a comprehensive resource for developing targeted effective therapies for rare disease patients.

The drawn-out traditional de novo drug development process, the success and high potential of computational drug repositioning, and the strong demand and the need to treat cancer, infectious, orphan, and rare diseases have, therefore, motivated researchers from different disciplines to unify forces in searching for therapeutic potentials and novel indications for existing drugs, which have already been approved for human use and are safer than products that are still being developed to treat cancer and other incurable diseases. Moreover, approved drugs are already optimized to target specific proteins, which could be highly useful if there is another disease that shares the same targets. Lastly, utilizing different sources of biological and biomedical data in developing computational drug repositioning models could be a promising tactic towards personalized treatment. Table 3 provides examples of drug repositioning applications in various disease areas and related therapeutics retrieved from Drugs@FDA database [91] and DrugBank [82].

## Drug repositioning opportunities

Drug repositioning is a highly promising technique that has attracted growing attention from governments and pharmaceutical companies for its key role in reducing time, cost, and risk in the process of developing drugs for cancers and other incurable illnesses. As this technique emerged, teams of multidisciplinary researchers and scientists carried out numerous attempts, with different degrees of efficiency and success, to computationally study the potential of repositioning drugs to treat other diseases and identify alternative indications regardless of the status of the investigated drug, whether it is approved, withdrawn, in clinical trials, or failed in clinical trials. Although drug repositioning is a quite up-and-coming technique, the traditional, costly, failure-prone de novo drug development process is still essential for discovering and testing new drugs; however, adopting some computational drug repositioning models within this process can help to push drugs steps forward in the development pipeline and eventually improve drug efficiencies in clinical trials.

The opportunities provided by drug repositioning to develop the urgently needed drugs to treat the current coronavirus epidemic cannot be underestimated. The general search for coronavirus effective drugs is reviewed on a weekly basis by Nature Medicine, the latest of which is [161]. The specific opportunites for repurposing drugs for coronavirus infections are reviewed in [5].

## Discussion and conclusion

After surveying various avenues in which computational drug repositioning strategies have been adopted, and models have been introduced to identify novel therapeutic interactions, we can conclude that each strategy and approach has its advantages and limitations and also that combining different strategies and approaches often achieve a higher success rate.

Despite having some outstanding computational drug repositioning models, developing robust models is still a complex process that comes with a few challenges. One of the main challenges is the difficulty of putting theoretical computational approaches into action; because of the complexity of mapping such theoretical approaches to simulate living organism’s behaviour and other obstacles such as missing, biased, and inaccurate data. For instance, reliable gene expression signature profiles may be hard to define due to several reasons such as variations in experimental conditions (e.g., environment variables and patient age) across different experiments, which may result in a data discrepancy in gene expression signatures, contributing to having biased data. Also, there may not always be significant changes in gene expressions when these genes are used as drug targets, which can lead to having inaccurate data. Moreover, the lack of high-resolution structural data for drug targets makes it hard to identify potential drug-target interactions when following the chemical structure and molecule information strategy. Another challenge facing computational drug repositioning models is the lack of trusted gold-standard datasets that can be used to evaluate the performance of such models.

Researchers, therefore, either have to build their own gold-standard dataset and subsequently use prevalent evaluation metrics (e.g., accuracy, recall, sensitivity, specificity, F1 score, and area under the receiver operating characteristic curve) to compare and evaluate their proposed models or they have to split their data into training, testing, and validating sets and then utilize K-fold cross-validation and prevalent evaluation metrics combined to avoid ending up with an over-fitted model.

Despite all the challenges encountered in computational drug repositioning studies, we envision that integrating multi-source data related to drugs (e.g., chemical structures), diseases (e.g., phenotypic information), and how these drugs and diseases affect human body (e.g., gene expression signature profiles and side effects) is crucial to enrich computational drug repositioning models and improve their performance and thus take them up to the next level. Furthermore, there is a significant number of diseases that still lack treatments to slow, stop, or reverse their courses, which motivates and inspires multidisciplinary researchers and scientists to carry out studies, especially in compacting different cancers and thousands of orphan and rare diseases.

In summary, we strongly believe that computational drug repositioning can be of enormous benefit to humanity by discovering new indications for approved drugs, speeding up the process of developing new drugs, and giving a second chance to withdrawn and failed drugs. While governments and pharmaceutical companies are directing more support towards computational drug repositioning ventures, researchers and scientists should pick up the ball and make further efforts to come up with creative state-of-the-art models towards novel findings and significant breakthroughs.

## Data Availability

No data to share as this is a review paper.
